# Daytime Sleepiness and Dispositional Optimism Are Related to Awake Bruxism Among Patients With Painful Temporomandibular Disorders

**DOI:** 10.1111/joor.70141

**Published:** 2025-12-25

**Authors:** Bachar Reda, Frank Lobbezoo, Daniele Manfredini, Maurits K. A. van Selms

**Affiliations:** ^1^ Department of Medical, Surgical and Health Sciences, School of Dentistry University of Trieste Trieste Italy; ^2^ Department of Orofacial Pain and Dysfunction, Academic Centre for Dentistry Amsterdam (ACTA) University of Amsterdam and Vrije Universiteit Amsterdam Amsterdam the Netherlands; ^3^ Department of Orofacial Pain and Jaw Function, Faculty of Odontology Malmö University Malmö Sweden; ^4^ Department of Medical Biotechnologies, School of Dentistry University of Siena Siena Italy

**Keywords:** awake bruxism, daytime sleepiness, dispositional optimism, oral behaviours, standardised tool for the assessment of bruxism (STAB)

## Abstract

**Objective:**

Awake bruxism (AB) is a multifactorial condition that has been linked to several physiological and psychological factors. The aim of this study was to investigate the potential association between AB‐related behaviours, daytime sleepiness, and dispositional optimism.

**Methods:**

A cross‐sectional study was conducted at the Academic Centre for Dentistry Amsterdam (ACTA). Participants with pain‐related temporomandibular disorders (TMD) that worsened with function were included. The survey comprised subjective assessment of the frequency of AB‐related oral behaviours (items adopted from the Standardised Tool for the Assessment of Bruxism, STAB), anxiety and depression (Patient Health Questionnaire‐4, PHQ‐4), daytime sleepiness (Epworth Sleepiness Scale, ESS), and dispositional optimism (Revised Life Orientation Scale, LOT‐R). Spearman's rank correlation and Mann–Whitney U tests were used for bivariate analysis. Ordinal logistic regression was conducted to determine the associations between AB behaviours, ESS and LOT‐R while controlling for age, sex, smoking status, anxiety and depression.

**Results:**

A total of 1661 patients were included, with a mean age of 42 years, of whom 77.6% were women and 84.2% were nonsmokers. Bivariate analyses showed significant associations between the frequency of AB‐related oral behaviours, ESS and LOT‐R. In the regression models, daytime sleepiness was found to be associated with increased frequency of teeth grinding, clenching and contact, but not with mandible bracing. Dispositional optimism was associated with a lower frequency of teeth grinding. Although statistically significant, the observed associations reflected small effect sizes.

**Conclusion:**

This study demonstrates an association, albeit with a small effect size, between the frequency of certain self‐reported AB behaviours and both daytime sleepiness and dispositional optimism. These findings suggest that, while these factors may play a modest role, they deserve consideration as potential contributors to the report of AB.

## Graphical abstract

Daytime sleepiness and dispositional optimism are related with awake bruxism among patients with painful temporomandibular disorders.

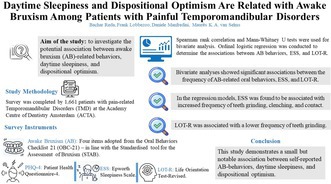

**Abbreviations**
AB: Awake Bruxism
ACTA: Academic Centre for Dentistry Amsterdam
DC/TMD: Diagnostic Criteria for Temporomandibular Disorders
EMA: Ecological Momentary Assessments
EMG: Electromyography
ESS: Epworth Sleepiness Scale
ETC‐ACTA: Ethical Committee of the Academic Centre for Dentistry Amsterdam
GAD‐7: Generalised Anxiety Disorder‐7
LOT‐R: Revised Life Orientation Scale
OBC‐21: Oral Behaviour Checklist‐21
OHRQoL: Oral Health‐Related Quality of Life
PHQ‐4: Patient Health Questionnaire‐4
PHQ‐9: Patient Health Questionnaire‐9
PSG: Polysomnography
SB: Sleep Bruxism
STAB: Standardised Tool for the Assessment of Bruxism
TMD: Temporomandibular Disorder
TMJ: Temporomandibular Joint

## 1 Introduction

Bruxism is defined by an international consensus of experts as ‘a repetitive jaw‐muscle activity characterized by clenching or grinding of the teeth, and/or bracing or thrusting of the mandible’ [1]. It can occur during both wakefulness and sleep, and is therefore classified as awake and sleep bruxism. Although numerous studies have reported its prevalence rate [2, 3, 4], variability in assessment methods, along with the use of ‘bruxism’ as an umbrella term without distinguishing specific activities such as teeth clenching or grinding, limits the accuracy of these estimates.

The aetiology of bruxism is multifactorial and has proven its association with several psychopathological symptoms, including stress, anxiety and depression [5, 6, 7, 8, 9, 10]. Other contributing factors include genetics and certain neurological conditions [11, 12, 13, 14], particularly in the neural pathways responsible for controlling jaw‐closing muscles. Additionally, disturbances in catecholamine levels, especially dopamine, which affects jaw muscle control, may result in bruxism development [14, 15]. Additionally, certain centrally acting drug classes, including phenethylamines, have been suggested to aggravate bruxism; however, further studies are required to establish a definitive causal relationship [16]. Both sleep bruxism (SB) and awake bruxism (AB) may result in prosthodontic complications, headache and reduced overall oral health‐related quality of life (OHRQoL) [14, 17, 18]. Additionally, excessive bruxism‐related masticatory muscle activities can be a risk factor for musculoskeletal symptoms affecting the temporomandibular joint (TMJ) and masticatory muscles [19, 20, 21, 22, 23], with jaw bracing reportedly being twofold more frequent in TMD patients compared to healthy individuals [24]. SB and AB have also been linked to poor subjective sleep quality [25].

Instrumental evaluation of bruxism using electromyography (EMG) and polysomnography (PSG) is considered the theoretical gold standard for bruxism assessment [26, 27]. However, it is less likely to be performed due to its limited feasibility related to its higher costs, the need for specialised equipment and advanced software analysis [28, 29, 30]. Common self‐report methods include yes/no questions about bruxism‐related behaviours, a reliable shortened six‐item version of the Oral Behaviour Checklist‐21 (OBC‐21) covering key behaviours within the bruxism spectrum [9], and ecological momentary assessment (EMA) [31, 32, 33]. To address the need for a comprehensive and multidimensional approach, the Standardised Tool for the Assessment of Bruxism (STAB) was recently developed [34]. The STAB combines self‐reported data, clinical examination and instrumental findings to evaluate bruxism status and its alleged consequences (Axis A), along with risk factors, etiological aspects and comorbid conditions (Axis B). A recent study, assessing self‐reported AB based on four OBC items adopted from the STAB, found an association between AB‐related oral behaviours and anxiety and depression [35].

Daytime sleepiness, while potentially attributable to factors such as sleep apnoea, disruption in circadian rhythm or central disorders [36], is also known to be associated with psychological distress [37], which in turn is a well‐known risk factor for the report of AB [9]. In this context, daytime sleepiness may reflect an underlying psychological strain that may be relevant to AB‐related behaviours. Although this suggests a potential indirect relationship between daytime sleepiness and AB, no studies have examined this potential association. This also accounts for dispositional optimism, a personality trait associated with positive expectations about the future. Among others, a general tendency to expect positive outcomes of life has been shown to buffer stress and enhance coping mechanisms [38, 39], which may, in turn, reduce the likelihood of engaging in AB‐related oral behaviours. Despite its potential relevance to AB, no study has investigated whether dispositional optimism could serve as a protective mechanism in the development of AB.

Thus, the aim of this study was to assess the frequency of AB‐related oral behaviours in relation to daytime sleepiness and dispositional optimism among patients with painful TMD using a series of items adopted from the STAB, while accounting for age, sex, smoking status, anxiety and depression.

## 2 Materials and Methods

### 2.1 Study Design, Settings and Recruitment

A cross‐sectional study was conducted between September 2014 and April 2023 at the Clinic for Orofacial Pain and Dysfunction at the Academic Centre for Dentistry Amsterdam (ACTA). The study population included adult patients (≥ 18 years) with painful TMD based on self‐report. These patients were recruited from those referred to the clinic for evaluation of orofacial complaints, including oral movement disorders (e.g., teeth grinding or clenching), orofacial pain (including pain‐related TMD), sleep apnoea or dental wear. All referred patients were assessed using a digital questionnaire incorporating screening tools from the Axis II protocol of the Diagnostic Criteria for Temporomandibular Disorder (DC/TMD) [40]. Only adult patients with self‐reported TMD pain who provided consent for the use of their data in research were included.

Patients were excluded if they: (1) were under 18 years of age; (2) did not report TMD‐related pain based on negative responses to two DC/TMD symptom‐based filter questions [40] (‘Have you ever had pain in the temporal region, jaw, in front of the ear, or inside the ear on either side’? and ‘In the last 30 days, which of the following best describes any pain in the temporal region, jaw, in front of the ear, or inside the ear on either side’?); (3) did not report pain worsening or relief in the temple, jaw, in front of the ear, or inside the ear on either side upon engaging with specific activities, including chewing tough or hard food, jaw movements (forward, sideways or opening the mouth), jaw habits such as holding teeth together, as clenching/grinding teeth, or chewing gum, or other jaw activities (talking, kissing or yawning); or (4) had incomplete questionnaire data.

### 2.2 Survey Instrument

After providing consent to participate, participants were required to complete a comprehensive questionnaire, also covering demographics (age, sex and smoking status), frequency of AB‐related oral behaviours, anxiety and depression, daytime sleepiness and dispositional optimism.

#### 2.2.1 AB Assessment

AB was assessed using four items of the OMC, in line with the subjective‐based assessment on AB according to the STAB [34, 41, 42]. Participants reported the frequency of engaging in each behaviour during waking hours over the last month, with answers ranging from ‘none of the time’ to ‘all of the time’. The four AB‐related oral behaviours were teeth grinding, teeth clenching, teeth contact and mandible bracing. Specifically, those behaviours correspond to the following OBC questions and STAB items:

OBC‐3/STAB A2.1: ‘In the last month, how often do you grind your teeth together during waking hours’?

OBC‐4/STAB A2.2: ‘In the last month, how often did you clench your teeth together during waking hours’?

OBC‐5/STAB A2.3: ‘In the last month, how often did you pressed, touched, or held teeth together other than while eating’?

OBC‐6/STAB A2.4: ‘In the last month, how often did you held, tightened, or tensed muscles without clenching or bringing teeth together’?

#### 2.2.2 Anxiety and Depression Assessment

In line with the STAB [34], anxiety and depression were assessed using two items from the Generalised Anxiety Disorder‐7 (GAD‐7) [43, 44], and two questions were adopted from the Patient Health Questionnaire‐9 (PHQ‐9) [45, 46]. Answers were ‘not at all’ granting the participant 0 points, ‘several days; 1 point’, ‘more than half of the days; 2 points’ and ‘nearly every day; 3 points’. The total PHQ‐4 score was obtained by summing the points for each question (0–12).

The Anxiety questions adopted from the GAD‐7 were as follows:

1. ‘Over the last two weeks, how often have you felt nervous, anxious, or on edge’?
2. ‘Over the last two weeks, how often were you unable to stop or control worrying’?

The Depression items adopted from the PHQ‐9 were as follows:

1. ‘In the last two weeks, how often you experienced little interest or pleasure in doing things’?
2. ‘In the last two weeks, how often you felt down, depressed, or hopeless’?

#### 2.2.3 Daytime Sleepiness Assessment

The validated Epworth Sleepiness Scale (ESS) was used to assess daytime sleepiness among study participants [47, 48]. Participants rated the likelihood to fall asleep during seven different situations. Answers included ‘would never nod off; 0 points’, ‘slight chance of nodding off; 1 point’ ‘moderate chance of nodding off; 2 points’ and ‘high chance of nodding off; 3 points’. Total ESS scores range from 0 to 24.

#### 2.2.4 Dispositional Optimism Assessment

Dispositional optimism, or the general tendency to expect positive outcomes of life, was evaluated using the revised version of the Life Orientation Test (LOT‐R) [49, 50]. LOT‐R comprises 10 questions with answers ‘strongly disagree; 0 points’, ‘disagree; 1 point’, ‘neutral; 2 points’, ‘agree; 3 points’ and ‘strongly agree; 4 points’. To calculate the total score, four items (questions number 2, 5, 6 and 8) are filler questions and were excluded from scoring. Items number 3, 7 and 9 are reverse‐coded using the formula: (Number of scale points + 1)—Respondents’ answer. The total score ranges from 0 to 24, with higher scores indicating a more optimistic view of the future and lower scores suggesting greater pessimism.

### 2.3 Ethical Considerations

The study protocol was approved by the ETC‐ACTA under protocol number 2024–58506. This study is not within the scope of the Medical Research Involving Human Subjects Act (WMO); thus, additional approval was not required. Only data from patients who provided consent for the use of their anonymised data for research purposes were included.

### 2.4 Statistical Analysis

Descriptive statistics were used to summarise participant characteristics. Continuous variables were reported as means and standard deviations, while categorical and ordinal variables were summarised using frequencies and percentages. Bivariate analyses were conducted to explore associations between the ordinal dependent variables and continuous predictors using Spearman's rank correlation, and between ordinal outcomes and binary predictors (sex and smoking) using the Mann–Whitney U test. Ordinal logistic regression was conducted for each individual AB‐related oral behaviour to assess associations with age, sex, smoking status, PHQ‐4, ESS and LOT‐R scores. Analyses were performed using SPSS version 26 (IBM Corp., Armonk, NY, USA), and statistical significance was set at *p* < 0.05.

## 3 Results

**FIGURE 1:**
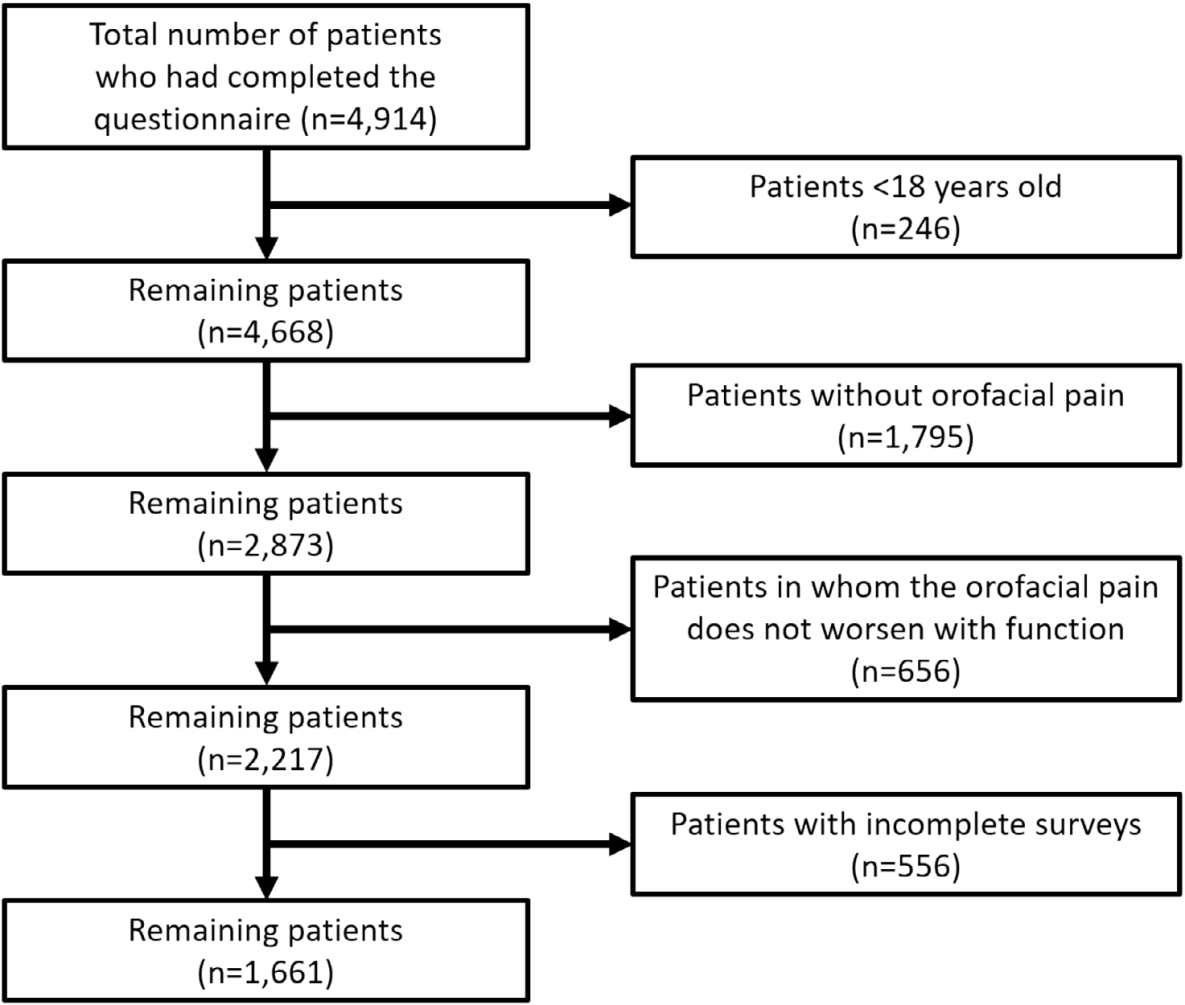
Flowchart of the study participants.

Even though a total of 4914 patients had completed the survey, only 1661 patients were included after applying the exclusion criteria (Figure 1). The mean age of the study population was 42.5 (± 15.3) years, and the majority were women (77.6%) and nonsmokers (84.2%).

Table 1 presents the frequency of engagement in AB‐related oral behaviours. Based on self‐reported responses, teeth grinding was the least commonly reported behaviour, with 61% of participants reporting no engagement during waking hours. For the psychological assessment tools, the mean scores were 3.0 (± 2.9) for PHQ‐4, 5.5 (± 4.3) for ESS, and 15.6 (± 4.6) for LOT‐R.

**TABLE 1:** Self‐reported frequency of awake bruxism‐related oral behaviours among participants.

	None of the time	A little of the time	Some of the time	Most of the time	All of the time
Teeth grinding	60.9% (*n* = 1011)	17.2% (*n* = 285)	12.9% (*n* = 214)	6.9% (*n* = 115)	2.2% (*n* = 36)
Teeth clenching	22.3% (*n* = 371)	15.8% (*n* = 262)	26.2% (*n* = 436)	29.3% (*n* = 487)	6.3% (*n* = 105)
Teeth contact	16.3% (*n* = 270)	16.5% (*n* = 274)	26.0% (*n* = 432)	33.2% (*n* = 552)	8.0% (*n* = 133)
Mandible bracing	26.7% (*n* = 443)	17.3% (*n* = 288)	22.9% (*n* = 381)	26.0% (*n* = 432)	7.0% (*n* = 117)

Abbreviation: *n*, number of participants.

Bivariate analyses indicated statistically significant associations between the frequency of all four AB‐related oral behaviours and smoking status, PHQ‐4, ESS and LOT‐R (*p* < 0.05). Age was significantly associated with teeth contact and mandible bracing, while sex was associated with teeth grinding and teeth clenching (*p* < 0.05). Detailed results are presented in Table 2.

**TABLE 2:** Bivariate analyses of awake bruxism‐related oral behaviours and independent variables.

	Independent variable	Test used	*p*
Teeth grinding	Age	Spearman's rank correlation	0.958
	Sex	Mann–Whitney U test	**0.036**
	Smoking status	Mann–Whitney U test	**0.001**
	PHQ‐4	Spearman's rank correlation	**< 0.001**
	ESS	Spearman's rank correlation	**< 0.001**
	LOT‐R	Spearman's rank correlation	**< 0.001**
Teeth clenching	Age	Spearman's rank correlation	0.449
	Sex	Mann–Whitney U test	**0.010**
	Smoking status	Mann–Whitney U test	**< 0.001**
	PHQ‐4	Spearman's rank correlation	**< 0.001**
	ESS	Spearman's rank correlation	**< 0.001**
	LOT‐R	Spearman's rank correlation	**< 0.001**
Teeth contact	Age	Spearman's rank correlation	**0.030**
	Sex	Mann–Whitney U test	0.953
	Smoking status	Mann–Whitney U test	**< 0.001**
	PHQ‐4	Spearman's rank correlation	**< 0.001**
	ESS	Spearman's rank correlation	**< 0.001**
	LOT‐R	Spearman's rank correlation	**< 0.001**
Mandible bracing	Age	Spearman's rank correlation	**0.001**
	Sex	Mann–Whitney U test	0.083
	Smoking status	Mann–Whitney U test	**0.001**
	PHQ‐4	Spearman's rank correlation	**< 0.001**
	ESS	Spearman's rank correlation	**0.001**
	LOT‐R	Spearman's rank correlation	**< 0.001**

*Note:* Bold *p* values indicate statistical significance at *p* < 0.05.

Abbreviations: ESS, Epworth Sleepiness Scale; LOT‐R, Life Orientation Scale‐Revised; PHQ‐4, Patient Health Questionnaire‐4.

Multiple ordinal logistic regression analyses showed that the factor smoking (yes/no) was significantly associated with higher frequency of engagement in all AB‐related behaviours (*p* < 0.05), with odds increasing by 43% for teeth grinding (OR = 1.429), 61.4% for teeth clenching (OR = 1.614), 43.4% for teeth contact (OR = 1.434), and 30% for mandible bracing (OR = 1.300). Women were associated with a greater likelihood of engaging in teeth clenching and mandible bracing by 38.5% and 26%, respectively (*p* < 0.05, OR = 1.385 and 1.261), but not with teeth grinding or teeth contact. Increased age by 1 year was only associated with a lower frequency of mandible bracing (*p* = 0.004), although the effect size was minimal (OR = 0.991). PHQ‐4 scores were associated with an increased frequency of engagement in the AB‐related oral behaviours (*p* < 0.001), with an increase in score with one point corresponding to a 10.4% increase in the frequency of teeth grinding (OR = 1.104), 14.6% in teeth clenching (OR = 1.146), 12% in teeth contact (OR = 1.119), and 15.4% in mandible bracing (OR = 1.154).

Similarly, higher ESS scores were associated with a more frequent engagement in AB‐related oral behaviours, except for mandible bracing. Specifically, each one‐point increase in ESS score (range 0–24) was associated with a 3.2% increase in the odds of more frequent teeth grinding (OR = 1.032), a 4.8% increase for teeth clenching (OR = 1.048), and a 4.3% increase for teeth contact (OR = 1.043). Higher LOT‐R scores were inversely associated with the frequency of teeth grinding (*p* < 0.05), with each one‐point increase (range 0–24) corresponding to a 2.5% decrease in the odds (OR = 0.975). While all associations were statistically significant, the observed effect sizes for daytime sleepiness and dispositional optimism were small. Detailed results of the multiple ordinal logistic regression are presented in Table 3.

**TABLE 3:** Multiple variable ordinal logistic regression analyses.

	Independent variable	Estimate	Odds ratio	*p*	95% confidence interval	
Teeth grinding	Age	0.003	1.003	0.439	−0.004	0.009
	Women	−0.199	0.819	0.086	−0.426	0.028
	Men	Reference				
	Smokers	0.357	1.429	**0.007**	0.099	0.614
	Nonsmoker	Reference				
	PHQ‐4	0.099	1.104	**< 0.001**	0.063	0.136
	ESS	0.032	1.032	**0.004**	0.010	0.054
	LOT‐R	−0.025	0.975	**0.039**	−0.048	0.001
Teeth clenching	Age	−0.001	1.001	0.823	−0.006	0.005
	Women	0.326	1.385	**0.002**	0.118	0.534
	Men	Reference				
	Smokers	0.479	1.614	**< 0.001**	0.236	0.721
	Nonsmokers	Reference				
	PHQ‐4	0.137	1.146	**< 0.001**	0.103	0.172
	ESS	0.047	1.048	**< 0.001**	0.027	0.068
	LOT‐R	−0.002	0.998	0.881	−0.23	0.020
Teeth contact	Age	−0.004	0.996	0.129	−0.010	0.001
	Women	0.036	1.036	0.732	−1.171	0.244
	Men	Reference				
	Smokers	0.361	1.434	**0.004**	0.118	0.604
	Nonsmokers	Reference				
	PHQ‐4	0.113	1.119	**< 0.001**	0.078	0.148
	ESS	0.043	1.043	**< 0.001**	0.022	0.063
	LOT‐R	0.005	1.005	0.652	−0.016	0.026
Mandible Bracing	Age	−0.009	0.991	**0.004**	−0.014	−0.003
	Women	0.232	1.261	**0.028**	0.024	0.439
	Men	Reference				
	Smokers	0.263	1.300	**0.032**	0.023	0.503
	Nonsmokers	Reference				
	PHQ‐4	0.144	1.154	**< 0.001**	0.110	0.179
	ESS	0.011	1.011	0.289	−0.009	0.031
	LOT‐R	−0.006	0.994	0.571	−0.027	0.015

*Note:* Bold *p* values indicate statistical significance at *p* < 0.05.

Abbreviations: ESS, Epworth Sleepiness Scale; LOT‐R, Life Orientation Scale‐Revised; PHQ‐4, Patient Health Questionnaire‐4.

## 4 Discussion

This study examined the frequency of AB‐related oral behaviours in relation to daytime sleepiness and dispositional optimism using items adopted from the STAB, while accounting for previously studied factors that were found to be associated with AB, including age, sex, smoking status, anxiety and depression.

Age was significantly associated with teeth contact and mandible bracing in the bivariate analysis, but only mandible bracing remained negatively associated with age after multivariable adjustment, indicating that older individuals tend to engage less frequently in this behaviour. Even though a previous study reported no association between AB and age [6], the assessment of bruxism was performed distinctively without considering the four dimensions of AB‐related behaviours.

Sex was associated with the frequency of awake teeth grinding and teeth clenching behaviours. When performing ordinal logistic regression, women were found to be more likely to engage in teeth clenching and mandible bracing compared to men. Our study results are in line with several previous studies reporting a higher prevalence of (awake) bruxism among women [51, 52]. The sex‐related differences in AB mandate the inclusion of this factor during clinical examinations as well as during research investigations.

Additionally, smoking status showed a positive relationship with the frequency of AB‐related behaviours, even after controlling for other factors in the regression analysis. Previous studies have reported the association between smoking and SB through objective polysomnography [53, 54]; however, its association with AB is still inconclusive. One recent study has found an association between mandible bracing and the number of years smoked among a non‐TMD population, but not with smoking status [55]. Another study stated the absence of a clear‐cut association between smoking and the frequency of AB behaviours among healthy individuals [35]. Differences in the clinical characteristics between our painful‐TMD sample and the non‐TMD populations in the other studies may partly explain the inconsistent findings. Biologically, smoking has demonstrated an increase in physiological arousal despite its stress‐relieving effect [56]. In addition, smoking increases the risk of depression, with smokers reporting higher levels of stress and depression compared to nonsmokers [57, 58]. Given that both stress and depression are established factors associated with the report of AB [6, 9], smoking may indirectly contribute to increased AB through these psychological pathways. Additionally, smoking has been associated with increased pain intensity among TMD patients [59], and both sleep and AB itself are linked with painful TMD [60]. Taken together, these overlapping factors support the need for a comprehensive objective assessment using EMG to determine the association between smoking and AB while accounting for possible confounding variables such as sex, psychological distress and the presence of TMD.

PHQ‐4 scores were significantly associated with the frequency of AB‐related oral behaviours in the bivariate analysis. Anxiety and depression remained significant positive predictors in the multiple ordinal regression models. These results corroborate the findings by van Selms and Lobbezoo, indicating a dose–response relationship between anxiety, depression and AB [9]. Moreover, a recent study employed the use of PHQ‐4 as adopted from the STAB and concluded that every 1% increase in the PHQ‐4 is associated with a fivefold increase in the frequency of AB‐oral behaviours [55]. These conditions have been reported to be associated with AB, supporting their inclusion in its assessment. In contrast, their association with SB remains unclear, which might be due to the greater complexity of SB‐related phenomena.

ESS scores were significantly associated with all four AB‐related oral behaviours in the bivariate analysis models. However, after controlling for possible confounding factors, daytime sleepiness was found to be associated with a significant increase in the reports of teeth grinding, teeth clenching and teeth contact, but not with mandible bracing. Although no studies have assessed the relationship between daytime sleepiness and AB, a previous study has shown that a poor quality of sleep, which can result in increased daytime sleepiness, was associated with AB [61]. In addition, obstructive sleep apnoea, one of the causes of daytime sleepiness, was found to be associated with SB [62, 63, 64]. Our study results indicate that daytime sleepiness, which can be due to sleep apnoea, disruption in circadian rhythm, or central disorders [36], seems to be associated with an increased frequency of engaging in AB‐related oral behaviours, suggesting a complex interplay of contributing factors. Although higher ESS scores are significantly associated with more frequent teeth grinding, clenching and contact, the effect sizes of the association are small, indicating that daytime sleepiness may have a limited influence on AB behaviours in this study population.

As for dispositional optimism (LOT‐R), it is associated with all four AB behaviours in the bivariate analysis (negative relationship) and emerged as a significant inverse predictor in the multiple ordinal regression model for teeth grinding only. This suggests that confounding variables such as smoking status, anxiety and depression may mask the true relationship between factors associated with AB in the unadjusted analysis. Our finding that higher optimism is linked to a lower frequency of awake teeth grinding might be explained by the fact that optimism is usually associated with better overall health, including mental health [65]. This suggests that patients with dispositional optimism are less susceptible to the adverse effects of psychological conditions and thus report less teeth grinding activities, although the observed effect size was small, indicating a limited practical impact.

This study illustrates the multifactorial etiological nature of AB, highlighting how demographic, behavioural and psychological factors jointly influence the report of AB. The fact that some psychological measures, such as dispositional optimism, lost their significant association with AB‐related oral behaviours except for teeth grinding after adjusting for confounders highlights the critical role of multivariable analysis in identifying true independent associations. Clinically, the assessment of AB should include evaluations of daytime sleepiness and dispositional optimism, alongside psychological conditions, demographic and lifestyle factors, to guide personalised management strategies. Addressing underlying contributing factors, such as excessive daytime sleepiness and pessimism, may help reduce AB indirectly. Moreover, the presence of AB may serve as a clinical indicator of broader underlying conditions, warranting further investigation and interdisciplinary care [66].

On the other hand, several limitations should be noted. All assessments, including AB frequency, were retrieved using self‐report, which may introduce recall bias and reduce accuracy compared to objective quantitative methods such as electromyography. This limitation is well acknowledged in the literature and highlights the relevance of ecological momentary assessment (EMA) as a more reliable alternative to single‐observation/recall self‐reported strategies. EMA captures behaviours in real time, thus reducing retrospective bias [31, 67, 68]. Given the small effect sizes observed for ESS and LOT‐R, the clinical relevance of these associations is uncertain. Larger and/or longitudinal studies are needed to better understand their impact on AB. The potential influence of undiagnosed sleep disorders, such as obstructive sleep apnoea, was not assessed and may represent an unmeasured confounding and mediating factor in the observed association between daytime sleepiness and AB behaviours. Moreover, the study sample consisted exclusively of individuals with signs and symptoms that were suggestive of painful TMD without clinical confirmation, thus potentially restricting the generalisability of findings. Future research should include more diverse populations with and without painful TMD, consider potential confounders, incorporate pain quality measures and use validated clinical criteria to confirm the presence of painful TMDs. Additionally, concurrent assessment of both awake and sleep bruxism should be considered, as evidence suggests that individuals with pain may exhibit increased masticatory muscle activity during both wakefulness and sleep [23].

## 5 Conclusion

This study highlights the association between AB‐related oral behaviours, daytime sleepiness and dispositional optimism, supporting the multifactorial nature of AB. Although the effect sizes for daytime sleepiness and dispositional optimism were small, their significant associations with AB suggest that clinical detection of AB should still prompt consideration of psychological and behavioural contributing factors to enhance evaluation and management.

## Author Contributions

Concept and design aspects were led by Bachar Reda and Maurits K. A. van Selms. Data acquisition and statistical analysis were conducted by Bachar Reda, Frank Lobbezoo, and Maurits K. A. van Selms. Analysis and interpretation of the data were performed collectively by Bachar Reda, Frank Lobbezoo, Daniele Manfredini and Maurits K. A. van Selms. Bachar Reda was responsible for drafting the manuscript, which was critically revised for important intellectual content by Frank Lobbezoo, Daniele Manfredini and Maurits K. A. van Selms. All authors reviewed and approved the final manuscript.

## Funding

The authors have nothing to report.

## Ethics Statement

The study protocol was approved by the Ethical Committee of the Academic Centre for Dentistry Amsterdam (ETC‐ACTA), under protocol number 2024–58506. This study is not within the scope of the Medical Research Involving Human Subjects Act (WMO); additional approval was not required.

## Consent

Only data from patients who provided consent for the use of their anonymised data for research purposes were included.

## Conflicts of Interest

The authors declare no conflicts of interest.

## Data Availability

The data that support the findings of this study are available from the corresponding author upon reasonable request.
